# Unsteady Radiative Natural Convective MHD Nanofluid Flow Past a Porous Moving Vertical Plate with Heat Source/Sink

**DOI:** 10.3390/molecules25040854

**Published:** 2020-02-14

**Authors:** Talha Anwar, Poom Kumam, Zahir Shah, Wiboonsak Watthayu, Phatiphat Thounthong

**Affiliations:** 1 Department of Mathematics, Faculty of Science, King Mongkut’s University of Technology Thonburi (KMUTT), 126 Pracha-Uthit Road, Bang Mod, Thrung Khru, Bangkok 10140, Thailand; anwartalha80@gmail.com (T.A.); wiboonsak.wat@kmutt.ac.th (W.W.); 2 KMUTT Fixed Point Research Laboratory, KMUTT-Fixed Point Theory and Applications Research Group, SCL 802 Fixed Point Laboratory, Department of Mathematics, Faculty of Science, King Mongkut’s University of Technology Thonburi (KMUTT), 126 Pracha-Uthit Road, Bang Mod, Thrung Khru, Bangkok 10140, Thailand; 3 Department of Medical Research, China Medical University Hospital, China Medical University, Taichung 40402, Taiwan; 4 Center of Excellence in Theoretical and Computational Science (TaCS-CoE), SCL 802 Fixed Point Laboratory, Science Laboratory Building, King Mongkut’s University of Technology Thonburi (KMUTT), 126 Pracha-Uthit Road, Bang Mod, Thrung Khru, Bangkok 10140, Thailand; zahir1987@yahoo.com; 5 Renewable Energy Research Centre, Department of Teacher Training in Electrical Engineering, Faculty of Technical Education, King Mongkut’s University of Technology North Bangkok, 1518 Pracharat 1 Road, Bangsue, Bangkok 10800, Thailand; phatiphat.t@fte.kmutnb.ac.th

**Keywords:** volume fraction, MHD, thermal radiation, porous medium, nanofluid, Laplace transform

## Abstract

In this research article, we investigated a comprehensive analysis of time-dependent free convection electrically and thermally conducted water-based nanofluid flow containing Copper and Titanium oxide (Cu and ${\mathrm{TiO}}_{2}$) past a moving porous vertical plate. A uniform transverse magnetic field is imposed perpendicular to the flow direction. Thermal radiation and heat sink terms are included in the energy equation. The governing equations of this flow consist of partial differential equations along with some initial and boundary conditions. The solution method of these flow interpreting equations comprised of two parts. Firstly, principal equations of flow are symmetrically transformed to a set of nonlinear coupled dimensionless partial differential equations using convenient dimensionless parameters. Secondly, the Laplace transformation technique is applied to those non-dimensional equations to get the close form exact solutions. The control of momentum and heat profile with respect to different associated parameters is analyzed thoroughly with the help of graphs. Fluid accelerates with increasing Grashof number (Gr) and porosity parameter (K), while increasing values of heat sink parameter (Q) and Prandtl number (Pr) drop the thermal profile. Moreover, velocity and thermal profile comparison for Cu and ${\mathrm{TiO}}_{2}$-based nanofluids is graphed.

## 1. Introduction

In recent times, nanotechnology is promptly influencing scientists and researchers for its significant role in industrial sciences. For instance, in the pharmaceutical field, patients of cancer are treated via nanoliquids based operators, comprises of different radiations and medicines. Some cooling and heating processes like minimizing receivable heat from computer processors, controlling the temperature of nuclear reactors, calming down the radiators in vehicles and handling of thermal flows in heat valves involve the nanoliquids. These key features, along with several industrial and domestic applications, nanofluids have fascinated investigators and scientists in modern days. Nanofluids immersed in regular fluids have a tendency to elevate their thermal performance.

A nanofluid consists of particles with a size scale in nanometers, named nanoparticles. This idea was initiated by Choi [1], when he dropped nano-sized solid particles in a base fluid, and called the new fluid a nanofluid. The formation of nanoparticles involves metals, carbides and carbon nanotubes. Nanoparticles have significant industrial applications such as sunscreens of vehicles are more resistive to radiations, bumpers of cars have lighter weight, synthetic bones are stronger, more stain repellent clothing and enhanced durability of balls for several sports. Extensively, in the age of nanotechnology, where every object is reducing in size and their features are enhancing, nano-catalysts have effective utility in various processes such as composite solid rocket propellants, purification of water, production of bio diesel, delivery of drugs and manufacturing of carbon nanotubes [2]. A nanofluid has a higher thermal conductivity in contrast to a regular fluid, because of additional thermal conductivity of combined nanoparticles, but certainly, it has relatively different structure as compared to regular fluid due to different sizes and shapes of nanoparticles [3]. Masuda et al. [4] presented that it is a characteristic of nanoparticles to elevate the thermal conductivity of fluids. It was found that the dispersion of carbon nanotubes in oil can enhance its thermal conductivity up to 50% [5]. Currently, the mangneto-nanofluids have become very significant due to the presence of favorable features regarding energy processes, materials engineering and medical operations. Many processes take place at very high temperature and preparation of equipment demands a deep knowledge of heat transfer. Satellites, space aircrafts, missiles, turbines and nuclear plants lie in the category of those processes. This fact draws the attention of many researchers to find such suitable combinations that have a maximum heat transfer rate. From the vast and different ranges of base fluid and nanoparticles, this work comprises of Copper (Cu) and Titanium oxide $\left({\mathrm{TiO}}_{2}\right)$ as nanoparticles and water as base fluid to find out the difference of heat enhancement and transfer rates.

The study of flows under the influence of many factors like magneto-hydrodynamics and thermal radiation is of deep concern due to its role in industries, physics, nuclear plants and chemical reactions. Das [6] provided a detailed analysis of natural convective flow along radiation effects for magneto-nanofluid. Das [7] investigated the motion and thermal behavior of nanofluids in a rotating frame. Ellahi et al. examined the importance of activation energy and chemical reaction for nanofluid peristaltic blood flow [8]. Theoretical aspects of unsteady magneto-hydrodynamics free convection flow were analyzed for some nanofluids by Hussanan et al. [9]. Ullah et al. [10] studied unsteady thin-film motion of nanofluid together with entropy generation. Wakif et al. [11] numerically analyzed the contribution of thermal radiation in time-dependent magneto-hydrodynamic (MHD) free convection couette flow of Cu-water with the help of single and two-phase models. Atif et al. [12] conducted a study to observe the impacts of Joule heating, viscous dissipation, internal heating and thermal radiation on MHD micro-polar Carreau nanofluid. Effects of the slip condition and magnetic field on natural convection in a vertical channel with water/alumina nanofluid were investigated by Malvandi et al. [13]. Mostafazadeh et al. [14] examined the influence of radiation on free convective laminar flow of nanofluid in a vertical enclosure employing single and two-phase models.

Another significant factor for fluid flow is heat generation/absorption. It has essential applications in the field of food industry, thermal engineering, mechanical engineering and physics like processes named as heat treatment, ventilation, and air conditioning. Food processing operations also involve cooling and heating processes [15]. Soomro et al. [16] investigated the heat generation/absorption and radiation effects on stagnation point flow of nanofluids. Heat generation/absorption effects on MHD free convection flow of a nanofluid were studied by Chamkha [17]. A detailed study covering the ion-slip and hall effect influence on CNTs along with heat control for porous surface was conducted by Ameen et al. [18]. Alzahrani et al. provided an in-depth analysis of heat consumption/generation of a Darcy flow for a rotating frame [19]. Hayat et al. investigated the flow and heat transfer behaviors for nanofluids in a rotating frame [20]. Performance of an Oldroyd-B fluid under the influence of thermal stratification and heat absorption/generation for mixed convection flow was examined by Hayat et al. [21]. Ebrahimi et al. [22] provided details of entropy generation and heat transfer in a micro-channel incorporating nanofluids. The influence of damped heat flux on natural convection flow of nanofluid past infinite vertical plate was studied by Nisa et al. [23].

In modern days, cavities filled with porous medium and fluid together are attracting the researchers and scientists. This kind of cavities has wide environmental and industrial utilities named nuclear fuel cooling, solidification, solar collectors, thermal insulation and so many others. These cavities can be divided either vertically [24,25,26] or horizontally [27,28,29]. A porous material means a medium whose structure has pores [30]. Umavathi [31] analyzed the characteristics of flow and heat transfer of composite porous medium saturated in nanofluid. Amhalhel et al. discussed the problems related to modeling of flow and heat transfer in porous medium [32]. Boundary layer flow of a permeable surface immersed in a porous medium regarding nanofluid was examined by Umar et al. [33]. Raju et al. [34] studied MHD flow of nanofluid over moving vertical plate in porous material under Soret and radiation impacts. AbdEl-Gaied et al. [35] reported the effect of a permeable moving flat plate on forced MHD laminar flow comprised of convective boundary conditions. Some other significant outcomes regarding thermal radiation and porous media were reported by [36,37,38,39,40].

The above literature review is the motivation behind the main emphasis of this article which is to examine the influence of porous material and heat sink on unsteady, MHD natural convection flow of nanofluid past a moving infinite vertical plate. The fluid motion occurs due to the impulsive movement of the plate and it is considered that flow is laminar. Water is considered as a base fluid and it contains two types of nanoparticles named Copper (Cu) and Titanium oxide $\left({\mathrm{TiO}}_{2}\right)$. The nonlinear function of thermal radiation is linearized with the aid of Taylor series and the closed-form solutions of modeled partial differential equations are derived by means of Laplace transformation. Furthermore, the influence of various pertinent parameters is illustrated through graphs.

## 2. Statement of Problem

**Figure 1:**
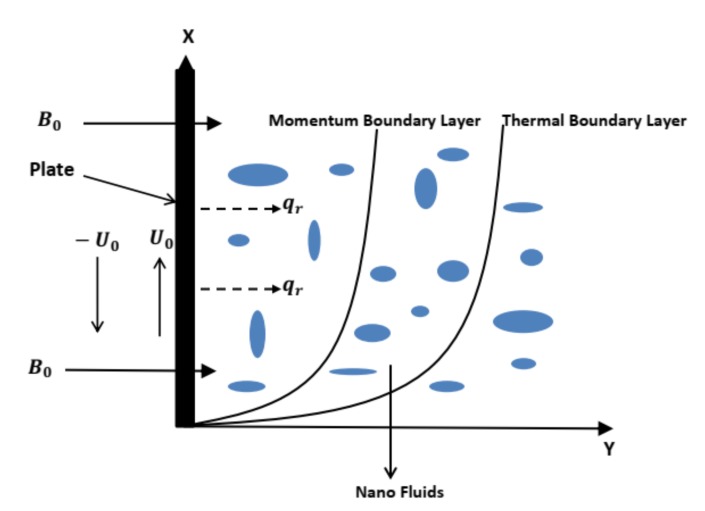
Presentation of geometry.

Suppose the natural convection based unsteady flow and shifting of heat for water-based nanofluid past a vertically infinite plate immersed in a porous material. Initially, the plate is static at $t^{*}=0$ with temperature $T_{\infty }^{*}$. Later on, the plate starts an instinctive motion with velocity $\lambda U_{0}$ in its own plane at $t^{*}>0$. Consequently, the temperature of the plate is enhanced or reduced to $T_{w}^{*}$. The considered geometry in the Cartesian plane is described as that *y*-axis is along the flow direction and *x*-axis is considered parallel to the plate. Moreover, the plate is saturated in a porous medium and it is assumed to be at $y^{*}=0$ and flow is restricted to $y^{*}>0$. As the vertical plate chosen in this work is infinitely long, temperature and velocity equations only depend on t and y. A uniform magnetic field of magnitude $B_{0}$ is acting along *y*-axis. The resulting magnetic field due to the flow of fluid along with the pressure gradient is neglected in contrast to the imposed magnetic field so that we consider the magnetic field as $B=(0,0,B_{0})$. This supposition is valid, since the magnetic Reynolds number is small enough for partially ionized fluids and metallic liquids [41]. Furthermore, to neglect the polarization effect of fluid, no external electric field is acting. It is assumed that in mass equation density is a linear function of thermal buoyancy forces. This assumption is sufficient for dropping both liquid and gases when temperature difference has small values. A radiative heat flux $q_{r}$ is also taken into account and it is considered that this heat flux in the x-direction is negligible against radiative heat flux in y-direction. A combination of base fluid water and nanoparticles named Copper (Cu) and Titanium Oxide $\left({\mathrm{TiO}}_{2}\right)$ is chosen and additionally, thermal equilibrium is assumed between these particles and base fluid water. It is also assumed that nanoparticles have a uniform size and shape. Lastly, a heat sink is also added to the considered system. The geometrical interpretation is provided in Figure 1.

In presence of all above assumptions, natural convection flow past a moving vertical plate embedded in porous medium incorporating heat sink, thermal radiation and magnetic field is presented by the given equations [42]

(1) $$\begin{array}{cc} \rho_{nf}\frac{\partial u^{*}}{\partial t^{*}} & =\mu_{nf}\frac{\partial^{2}u^{*}}{\partial y^{*2}}+g{\left(\rho \beta \right)}_{nf}\left(T^{*}-T_{\infty }^{*}\right)-\sigma_{nf}B_{0}^{2}u^{*}-\frac{\mu_{nf}}{k^{*}}u^{*}, \end{array}$$

(2) $$\begin{array}{cc} \;\;{\left(\rho c_{p}\right)}_{nf}\frac{\partial T^{*}}{\partial t^{*}} & =k_{nf}\frac{\partial^{2}T^{*}}{\partial y^{*2}}-\frac{\partial q_{r}}{\partial y^{*}}-Q_{0}\left(T^{*}-T_{\infty }^{*}\right), \end{array}$$

where $u^{*}$ is the velocity of fluid along the x-direction, $T^{*}$ is the temperature of fluid during flow, $\mu_{nf}$ is the dynamic viscosity, $\beta_{nf}$ is the thermal expansion coefficient, $\rho_{nf}$ is the density, $\sigma_{nf}$ is the electrical conductivity, $k_{nf}$ is the thermal conductivity, *g* is gravitational acceleration, $q_{r}$ is the radiative heat flux, ${\left(\rho c_{p}\right)}_{nf}$ is the heat capacitance and the term $Q_{0}$ with negative sign shows that a heat sink is added to system under observation.

The physical quantities $\mu_{nf},\rho_{nf},{\left(\rho c_{p}\right)}_{nf}$ and ${\left(\rho \beta \right)}_{nf}$ are deduced by manipulating the expressions provided by [43]

(3) $$\begin{array}{cc} \mu_{nf} & =\frac{\mu_{f}}{{\left(1-\phi \right)}^{2.5}},\;\rho_{nf}=\phi \rho_{s}+\left(1-\phi \right)\rho_{f},\;{\left(\rho c_{p}\right)}_{nf}=\phi {\left(\rho c_{p}\right)}_{s}+\left(1-\phi \right){\left(\rho c_{p}\right)}_{f},\\ {\left(\rho \beta \right)}_{nf} & =\phi {\left(\rho \beta \right)}_{s}+\left(1-\phi \right){\left(\rho \beta \right)}_{f},\;\sigma_{nf}=\left[\frac{3(\sigma -1)\phi }{(\sigma +2)-(\sigma -1)\phi }+1\right]\sigma_{f},\;\sigma =\frac{\sigma_{s}}{\sigma_{f}}. \end{array}$$

where ϕ is solid volume fraction of nanoparticle, $\rho_{f}$ is the density of base fluid, $\rho_{s}$ is the density of nanoparticle, $\sigma_{f}$ is the electrical conductivity of base fluid, $\mu_{f}$ is the dynamic viscosity of base fluid, ${\left(\rho c_{p}\right)}_{f}$ is the heat capacitance of base fluid, ${\left(\rho c_{p}\right)}_{s}$ is the heat capacitance of nanoparticle. To deal the thermal conductivity of nanofluid, model given by Hamilton and Crosser, followed by Kakac [44] and Oztop [45] is utilized as

(4) $$\begin{array}{c} k_{nf}=\left[\frac{k_{s}-2\phi \left(k_{f}-k_{s}\right)+2k_{f}}{k_{s}+\phi \left(k_{f}-k_{s}\right)+2k_{f}}\right]k_{f}, \end{array}$$

where $k_{s}$ and $k_{f}$ represent the thermal conductivity of nanoparticle and base fluid respectively.

The associated initial and boundary conditions of considered problem are presented as:

(5) $$\begin{array}{c} u^{*}\left(y^{*},0\right)=0,\;T^{*}\left(y^{*},0\right)=T_{\infty }^{*},\;\mathrm{for}\;y^{*}\geq 0,\\ u^{*}\left(y^{*},t^{*}\right)\to 0,\;T^{*}\left(y^{*},t^{*}\right)\to T_{\infty }^{*},\;\mathrm{for}\;y^{*}\to \infty ,\\ u^{*}\left(0,t^{*}\right)=\lambda U_{0},\;T^{*}\left(0,t^{*}\right)=T_{w}^{*},\;\mathrm{for}\;t^{*}>0, \end{array}$$

where λ=0 shows the static plate and λ=±1 represents the forth and back movement of the plate.

The radiation heat flux, after using Rosseland approximation comes out to be [46]

(6) $$\begin{array}{c} q_{r}=-\frac{4\sigma^{*}\partial T^{*4}}{3k_{1}\partial y^{*}}, \end{array}$$

where the Stafan–Boltzman constant and adsorption coefficient are represented by $\sigma^{*}$ and $k_{1}$, respectively.

The term $q_{r}$ can be linearized by expansion of $T^{*4}$ using Taylor series about $T_{\infty }^{*}$, keeping the supposition in mind that temperature differences are small enough to neglect the higher-order terms. After normalizing, $T^{*4}$ comes out to be $T^{*4}\approx 4T_{\infty }^{*3}T^{*}-3T_{\infty }^{*4}$.

On using this linearization in Equation () results in

(7) $$\begin{array}{c} {\left(\rho c_{p}\right)}_{nf}\frac{\partial T^{*}}{\partial t^{*}}=\left(k_{nf}+\frac{16\sigma^{*}T_{\infty }^{*3}}{3k_{1}}\right)\frac{\partial^{2}T^{*}}{\partial y^{*2}}-Q_{0}\left(T^{*}-T_{\infty }^{*}\right). \end{array}$$

The non-dimensional quantities are introduced as:

(8) $$\begin{array}{c} u=\frac{u^{*}}{U_{0}},\;y=\frac{y^{*}U_{0}}{\nu },\;t=\frac{t^{*}U_{0}^{2}}{\nu },\;\theta =\frac{T^{*}-T_{\infty }^{*}}{T_{w}^{*}-T_{\infty }^{*}}. \end{array}$$

On using above non-dimensional quantities, Equations (1) and (7) turn out to be as follows

(9) $$\begin{array}{cc} \frac{\partial u}{\partial t} & =a_{1}\frac{\partial^{2}u}{\partial y^{2}}-M^{2}a_{3}u+Gra_{2}\theta -\frac{a_{1}}{K}u, \end{array}$$

(10) $$\begin{array}{cc} \frac{\partial \theta }{\partial t} & =a_{4}\frac{\partial^{2}\theta }{\partial y^{2}}-a_{5}\theta .\;\; \end{array}$$

where

(11) $$\begin{array}{cc} x_{1} & =\left[\phi \frac{\rho_{s}}{\rho_{f}}-\phi +1\right],\;x_{2}=\left[\phi \frac{{\left(\rho \beta \right)}_{s}}{{\left(\rho \beta \right)}_{f}}-\phi +1\right],\\ x_{3} & =\left[\phi \frac{{\left(\rho c_{p}\right)}_{s}}{{\left(\rho c_{p}\right)}_{f}}-\phi +1\right],\;x_{4}=\left[\frac{k_{s}-2\phi \left(k_{f}-k_{s}\right)+2k_{f}}{k_{s}+\phi \left(k_{f}-k_{s}\right)+2k_{f}}\right],\\ x_{5} & =\left[1+\frac{3(\sigma -1)\phi }{(\sigma +2)-(\sigma -1)\phi }\right],\;x_{6}=\frac{x_{4}}{x_{3}},\;a_{1}=\frac{1}{{\left(1-\phi \right)}^{2.5}x_{1}},\\ \;a_{2} & =\frac{x_{2}}{x_{1}},\;a_{3}=\frac{x_{5}}{x_{1}},\;a_{4}=\frac{1}{x_{3}Pr}\left(x_{4}+Nr\right),\;a_{5}=\frac{Q}{x_{3}}, \end{array}$$

and non-dimensional parameters are defined as

(12) $$\begin{array}{c} Gr=\frac{g\beta_{f}\nu_{f}(T_{w}^{*}-T_{\infty }^{*})}{U_{0}^{3}},\;Nr=\frac{16\sigma^{*}T_{\infty }^{*3}}{3k_{f}k_{1}},\;M^{2}=\frac{\sigma_{f}B_{0}^{2}\nu_{f}}{\rho_{f}U_{0}^{2}},\\ \;Pr=\frac{\mu_{f}{\left(c_{p}\right)}_{f}}{k_{f}},\;\frac{1}{K}=\frac{\nu_{f}^{2}}{k^{*}U_{0}^{2}},\;Q=\frac{Q_{0}\nu_{f}}{{\left(\rho c_{p}\right)}_{f}U_{0}^{2}}.\;\;\; \end{array}$$

Here, the Grashof number is denoted by Gr, the radiation parameter is denoted by Nr, the magnetic parameter is denoted by $M^{2}$, the Prandtle number is denoted by Pr, the parameter of permeability is denoted by K and lastly Q is the heat sink parameter.

The associated initial and boundary conditions takes the following form after the introduction of dimensionless parameters:

(13) $$\begin{array}{c} u(y,0)=0,\;\theta (y,0)=0,\;\mathrm{for}\;y\geq 0,\; \end{array}$$

(14) $$\begin{array}{c} u(y,t)\to 0,\;\theta (y,t)\to 0,\;\mathrm{for}\;y\to \infty , \end{array}$$

(15) $$\begin{array}{c} u(0,t)=\lambda ,\;\theta (0,t)=1,\;\mathrm{for}\;t>0.\; \end{array}$$

## 3. Analytical Solution of Problem

To generate the solution of this problem, Laplace transform [47] is convenient tool because of non uniform boundary conditions. The other convenient methods like Adomian decomposition, homotopy analysis method, perturbation method and separation of variables do not serve the purpose here due to boundary conditions. we formulate the Laplace transform pair for the sake of results of current problem as an integral of the following form

(16) $$\begin{array}{c} \bar{R}\left(y,s\right)=\int_{0}^{\infty }e^{-st}R\left(y,t\right)dt=L\left[R\right]\left(t\right),\;\;t\geq 0, \end{array}$$

where R∈{u,θ}. The above integral is convergent for $Re\left(s\right)>\gamma_{0}$, and s=Ψ+jΩ, $\gamma_{o}$ is some positive real number and $j=\sqrt{-1}$.

Transformation of the Laplace domain solutions back to original time domain can be done such as

(17) $$\begin{array}{c} R\left(y,t\right)=\frac{1}{2\pi j}\int_{BR}e^{st}\bar{R}\left(y,s\right)ds=L^{-1}\left[\bar{R}\right]\left(s\right), \end{array}$$

On using Laplace transform, Equations (9) and (10) obtain the following form

(18) $$\begin{array}{cc} s\bar{u} & =a_{1}\frac{\partial^{2}\bar{u}}{\partial y^{2}}-\left(M^{2}a_{3}+a_{1}\frac{1}{K}\right)\bar{u}+Gra_{2}\bar{\theta }, \end{array}$$

(19) $$\begin{array}{cc} s\bar{\theta } & =a_{4}\frac{\partial^{2}\bar{\theta }}{\partial y^{2}}-a_{5}\bar{\theta }.\;\; \end{array}$$

The initial and boundary conditions in Laplace domain are gives as:

(20) $$\begin{array}{cc} \bar{u}\left(y,0\right) & =0,\;\bar{\theta }\left(y,0\right)=0, \end{array}$$

(21) $$\begin{array}{cc} \bar{u}\left(y,s\right) & \to 0,\;\bar{\theta }\left(y,s\right)\to 0,\;\mathrm{for}\;y\to \infty , \end{array}$$

(22) $$\begin{array}{cc} \bar{u}\left(0,s\right) & =\frac{\lambda }{s},\;\bar{\theta }\left(0,s\right)=\frac{1}{s}. \end{array}$$

The solution of Equations (18) and (19) according to Conditions (20)–(22) are evaluated as

(23) $$\begin{array}{cc} \bar{\theta }\left(y,s\right) & =\frac{1}{s}e^{-\sqrt{\alpha }\sqrt{s+a_{5}}y}, \end{array}$$

(24) $$\begin{array}{cc} \bar{u}\left(y,s\right) & =\frac{\lambda }{s}e^{-\sqrt{\beta }\sqrt{s+a^{*}}y}+\frac{Gra_{6}}{b}\left[\frac{1}{s-b}-\frac{1}{s}\right]\left(e^{-\sqrt{\beta }\sqrt{s+a^{*}}y}-e^{-\sqrt{\alpha }\sqrt{s+a_{5}}y}\right), \end{array}$$

where

(25) $$\begin{array}{c} \alpha =\frac{1}{a_{4}},\;\beta =\frac{1}{a_{1}},\;a^{*}=M^{2}a_{3}+a_{1}\frac{1}{K},\;b=\frac{a^{*}\beta -\alpha a_{5}}{\alpha -\beta },\;a_{6}=\frac{a_{2}\beta }{\alpha -\beta }.\; \end{array}$$

The implementation of inverse Laplace transform provides the following relation for velocity and temperature in real time domain

(26) $$\begin{array}{cc} \theta (y,t) & =f_{0}\left(y,\alpha ,a_{5},t\right), \end{array}$$

(27) $$\begin{array}{cc} u(y,t) & =\lambda f_{1}\left(y,\beta ,a^{*},t\right)+\frac{Gra_{6}}{b}\left[f_{2}\left(y,\beta ,a^{*},b,t\right)-f_{3}\left(y,\beta ,a_{5},b,t\right)-f_{1}\left(y,\beta ,a^{*},t\right)+f_{0}\left(y,\alpha ,a_{5},t\right)\right], \end{array}$$

with

(28) $$\begin{array}{cc} f_{0}\left(y,\alpha ,a_{5},t\right) & =\frac{1}{2}\left[e^{-y\sqrt{\alpha a_{5}}}\mathrm{erfc}\left(\frac{y\sqrt{\alpha }}{2\sqrt{t}}-\sqrt{a_{5}t}\right)+e^{y\sqrt{\alpha a_{5}}}\mathrm{erfc}\left(\frac{y\sqrt{\alpha }}{2\sqrt{t}}+\sqrt{a_{5}t}\right)\right],\\ f_{1}\left(y,\beta ,a^{*},t\right) & =\frac{1}{2}\left[e^{-y\sqrt{\beta a^{*}}}\mathrm{erfc}\left(\frac{y\sqrt{\beta }}{2\sqrt{t}}-\sqrt{a^{*}t}\right)+e^{y\sqrt{\beta a^{*}}}\mathrm{erfc}\left(\frac{y\sqrt{\beta }}{2\sqrt{t}}+\sqrt{a^{*}t}\right)\right],\\ f_{2}\left(y,\beta ,a^{*},b,t\right) & =\frac{e^{bt}}{2}\left[e^{-y\sqrt{\beta }\sqrt{b+a^{*}}}\mathrm{erfc}\left(\frac{y\sqrt{\beta }}{2\sqrt{t}}-\sqrt{(b+a^{*})t}\right)+\right.\\ \; & \left.e^{y\sqrt{\beta }\sqrt{b+a^{*}}}\mathrm{erfc}\left(\frac{y\sqrt{\beta }}{2\sqrt{t}}+\sqrt{(b+a^{*})t}\right)\right],\\ f_{3}\left(y,\beta ,a_{5},b,t\right) & =\frac{e^{bt}}{2}\left[e^{-y\sqrt{\alpha }\sqrt{b+a_{5}}}\mathrm{erfc}\left(\frac{y\sqrt{\alpha }}{2\sqrt{t}}-\sqrt{(b+a_{5})t}\right)+\right.\\ \; & \left.e^{y\sqrt{\alpha }\sqrt{b+a_{5}}}\mathrm{erfc}\left(\frac{y\sqrt{\alpha }}{2\sqrt{t}}+\sqrt{(b+a_{5})t}\right)\right], \end{array}$$

where the complementary error function is defined as

$$erfc(w)=\frac{2}{\sqrt{\pi }}\int_{w}^{\infty }e^{-z^{2}}dz.$$

The Nusselt number is given as follows:

(29) $$\begin{array}{cc} N_{u} & =\theta^{\prime }\left(0,y\right)=\frac{\partial \theta }{\partial y}\left(0,y\right),\\ N_{u} & =-\sqrt{\frac{\alpha }{\pi t}}e^{-a_{5}t}-\sqrt{\alpha a_{5}}\mathrm{erf}\left(\sqrt{a_{5}t}\right), \end{array}$$

where error function is defined as

$$\mathrm{erf}\left(w\right)=\frac{2}{\sqrt{\pi }}\int_{0}^{w}e^{-z^{2}}dz.$$

## 4. Numerical Case Studies

**Figure 2:**
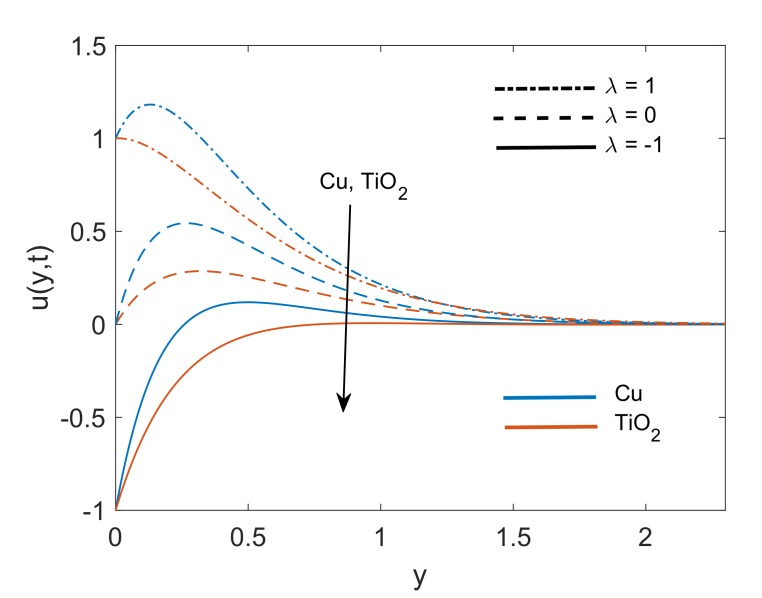
Velocity profile comparison for Cu-water and ${\mathrm{TiO}}_{2}$-water when $Gr=5,Nr=1.5,K=5,Q=0.5,M^{2}=5,\phi =0.1$ and t=0.5.

**Figure 3:**
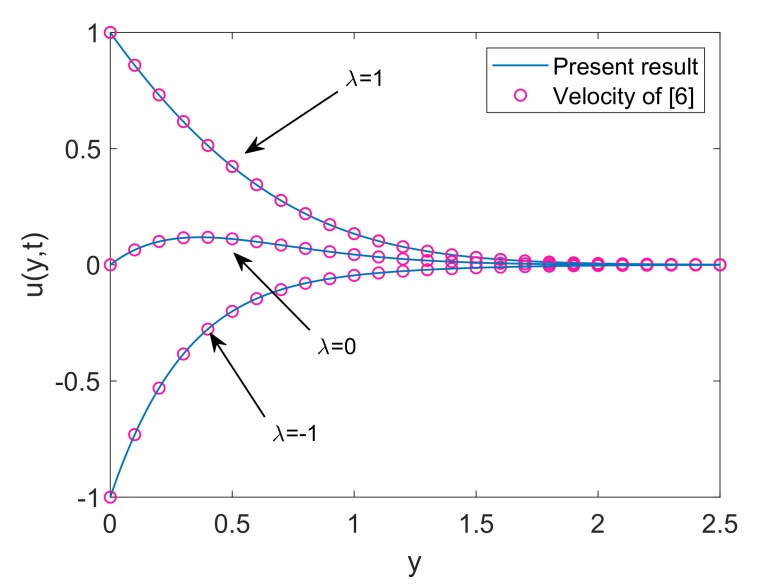
Velocity comparison between present work and Das [6] when $Gr=5,Nr=0.5,K=100,Q=0,M^{2}=5,\phi =0.1$ and t=0.5.

**Figure 4:**
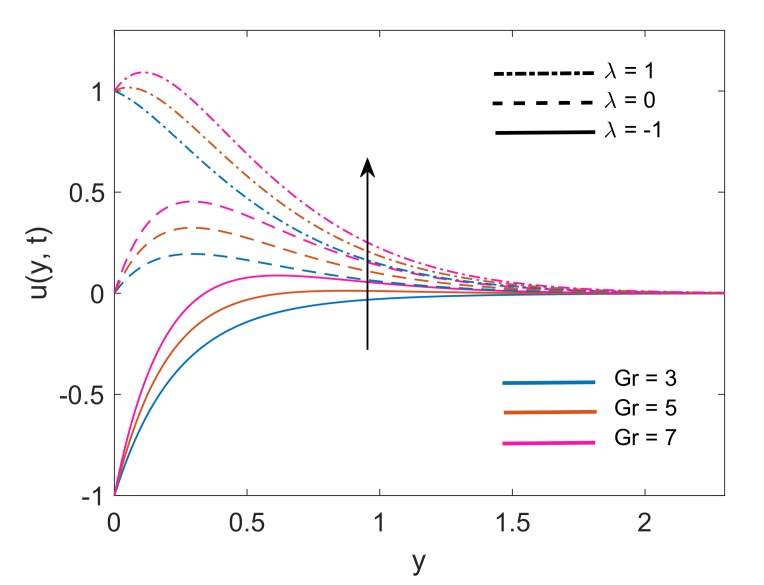
Variation in velocity profile for different Gr when $Nr=1.5,K=5,Q=0.5,M^{2}=5,\phi =0.1$ and t=0.5.

**Figure 5:**
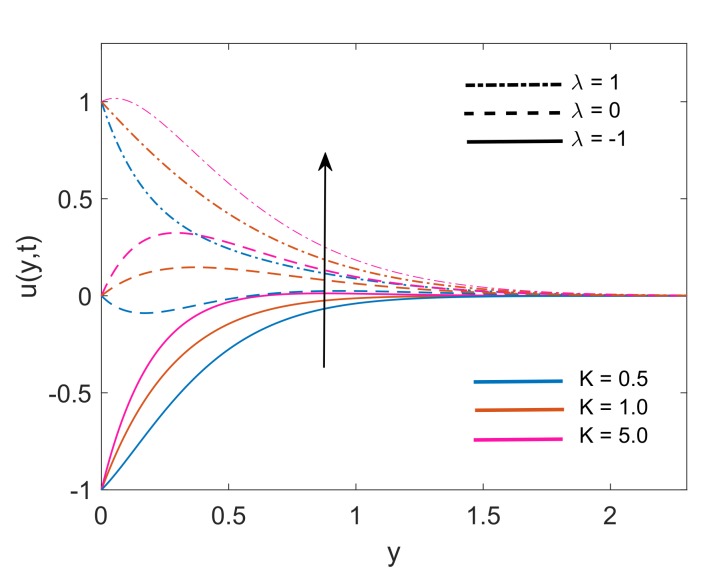
Variation in velocity profile for different *K* when $Nr=1.5,Gr=5,Q=0.5,M^{2}=5,\phi =0.1$ and t=0.5.

**Figure 6:**
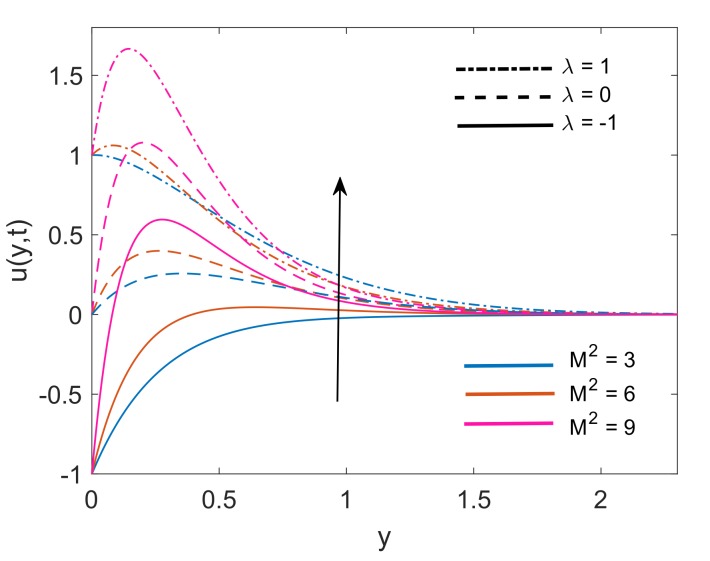
Variation in velocity profile for different $M^{2}$ when Nr=1.5,Gr=5,K=5,Q=0.5,ϕ=0.1 and t=0.5.

**Figure 7:**
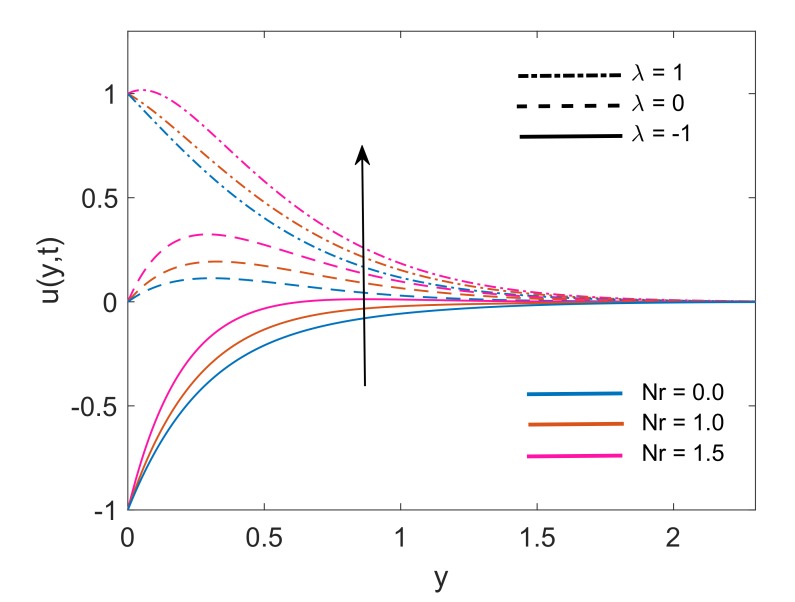
Variation in velocity profile for different Nr when $Gr=5,K=5,Q=0.5,M^{2}=5,\phi =0.1$ and t=0.5.

**Figure 8:**
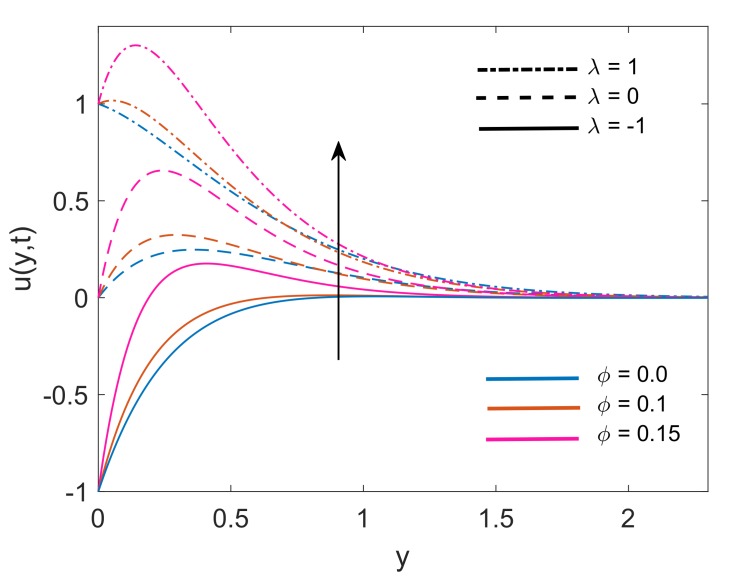
Variation in velocity profile for different ϕ when $Nr=1.5,Gr=5,K=5,Q=0.5,M^{2}=5$ and t=0.5.

**Figure 9:**
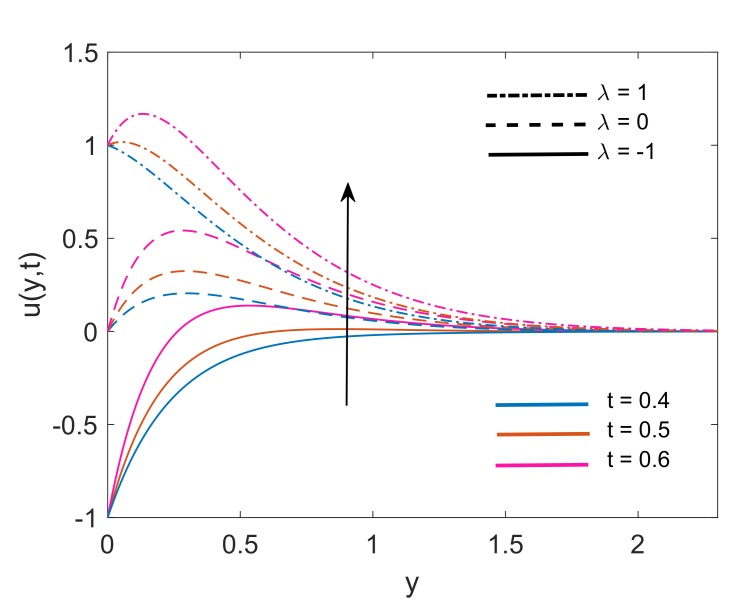
Variation in velocity profile for different *t* when $Nr=1.5,Gr=5,K=5,Q=0.5,M^{2}=5$ and ϕ=0.1.

**Figure 10:**
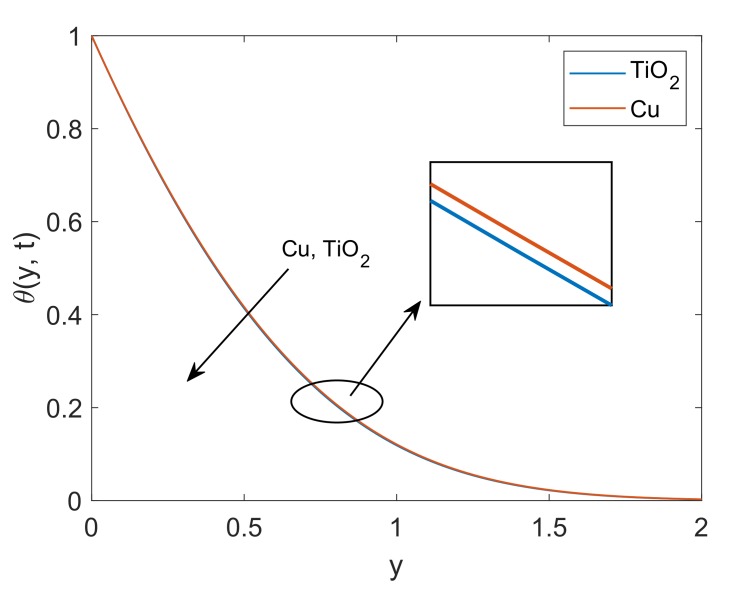
Temperature profile comparison for Cu-water and ${\mathrm{TiO}}_{2}$-water when Nr=5,Q=0.5,Pr=6.2,ϕ=0.1 and t=0.5.

**Figure 11:**
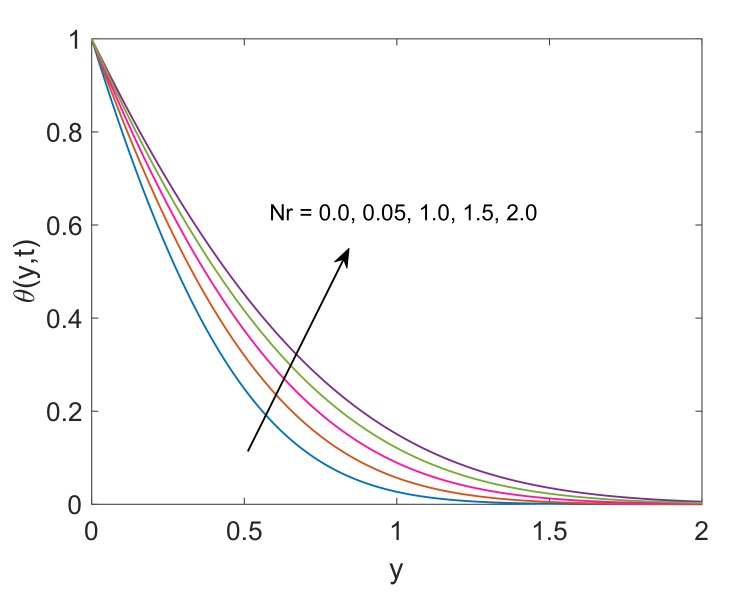
Temperature profile for different Nr when Q=0.5,Pr=6.2,ϕ=0.1 and t=0.5.

**Figure 12:**
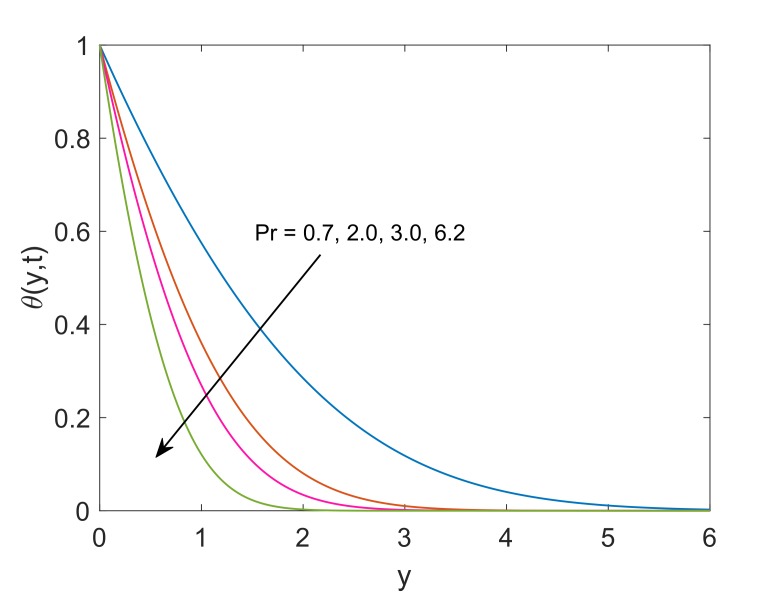
Temperature profile for different Pr when Q=0.5,Nr=1.5,ϕ=0.1 and t=0.5.

**Figure 13:**
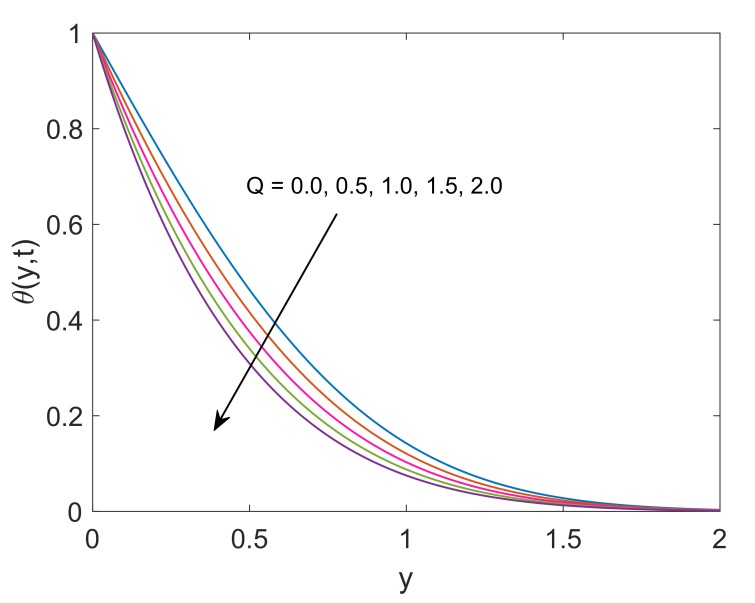
Temperature profile for different *Q* when Pr=6.2,Nr=1.5,ϕ=0.1 and t=0.5.

**Figure 14:**
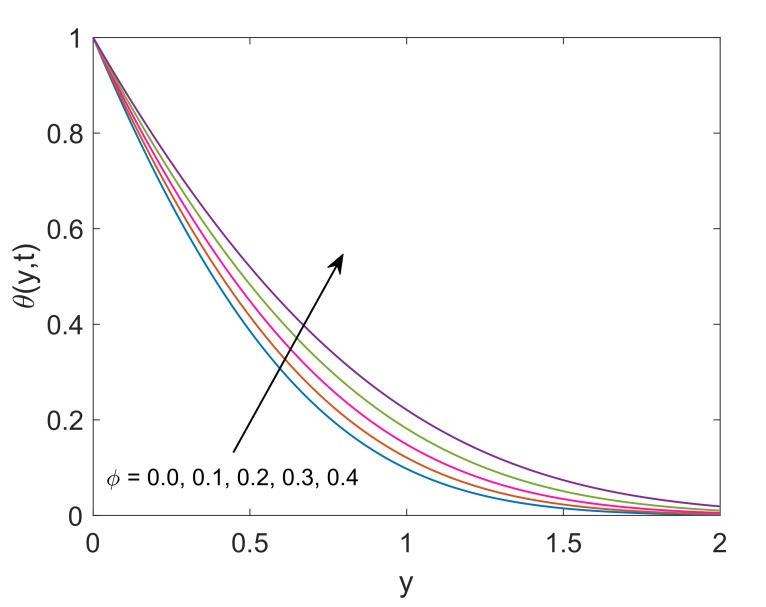
Temperature profile for different ϕ when Pr=6.2,Nr=1.5,Q=0.5 and t=0.5.

**Figure 15:**
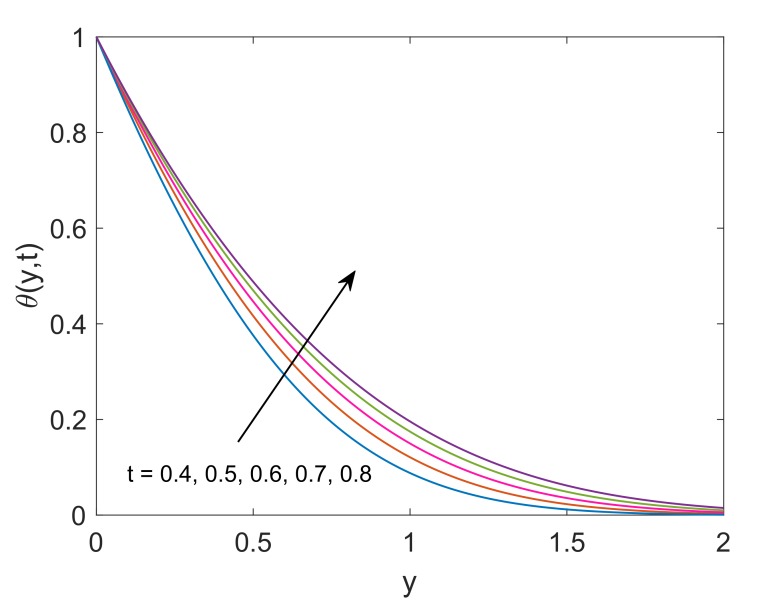
Temperature profile for different *t* when Pr=6.2,Nr=1.5,ϕ=0.1 and Q=0.5.

**Figure 16:**
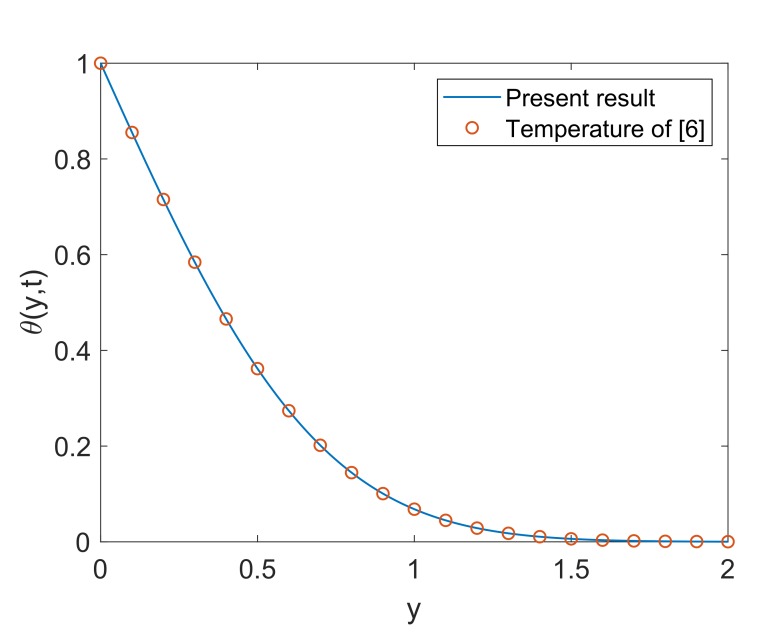
Temperature comparison between present work and Das [6] when Pr=6.2,Nr=0.5,Q=0,ϕ=0.1 and t=0.5.

To deeply understand the physics of the current problem, a parametric study is conducted and obtained outcomes are delineated with the help of graphs. The physical features of dimensionless fluid temperature and velocity as a result of variation in most significant substantial factors like Grashof number (Gr), magnetic parameter $\left(M^{2}\right)$, porosity parameter (K), radiation parameter (Nr), heat sink parameter (Q) and solid volume fraction of nanoparticles (ϕ) are presented in Figure 2, Figure 3, Figure 4, Figure 5, Figure 6, Figure 7, Figure 8, Figure 9, Figure 10, Figure 11, Figure 12, Figure 13, Figure 14, Figure 15 and Figure 16. The values of the volume fraction of nanoparticles belongs to the interval [0,0.2]. The case λ=1 corresponds to the upward motion of vertical plate, λ=−1 corresponds to the downward motion of the vertical plate and λ=0 for static plate. Moreover, Q=0,Nr=0 and ϕ=0 corresponds to the absence of heat sink parameter, radiation parameter and nanoparticles respectively.

Figure 2 presents the velocity profile of both nanofluids Cu-water and ${\mathrm{TiO}}_{2}$-water with the same volume fraction of both nanoparticles. It is revealed that Cu-water has a thinner boundary layer which results due to an increase in its dynamic viscosity because of the relatively higher density of Cu. From Figure 3, an exact agreement between velocity solution of current work and Das [6] can be observed for Q=0 and $\frac{1}{K}\to 0$. This agreement verifies the velocity solution for our current work. Figure 4 interprets the variation of velocity profile for various values of Gr. In the physical sense, Gr deals with the fraction of thermal buoyancy force to viscous force. Increase in Gr implies that buoyancy force together with the aid of allied forces is getting stronger and eventually it is suppressing the viscous forces. This factor justifies the decrease in resistance and ultimately fluid gets accelerated. A similar kind of behavior is witnessed for both λ=0 and λ=±1 as well. Furthermore, On free stream surface, away from the plate buoyancy force weakens and fluid attains the zero velocity.

Figure 5 demonstrates the velocity profile for variation in values of K. Respective Figure interprets that increasing the value of K leads to an increase in the thickness of the momentum boundary layer. Physical justification of this fact is that increment in K, decreases the resistance offered by a porous medium which in turn enhances the momentum development of the regime and consequently, the velocity of the fluid is increased. Enhancement in fluid velocity is spotted for both stationary (λ=0) and moving vertical plate (λ=±1). Figure 6 depicts the variation in velocity, when the strength of the imposed magnetic field is increased (i.e., $M^{2}$ increases). It is witnessed that dimensionless velocity has higher values when $M^{2}$ increases, for static plate (λ=0) and moving plate (λ=±1) as well. This enhancement in momentum boundary layer thickness is certified by the physical fact that when the magnetic force lines past the vertical plate, they give a sudden push to decelerated fluid and as a result fluid overcomes the viscous forces. Consequently, the velocity of the fluid faces a rise when the value of $M^{2}$ increases. This sudden push is featured by instinctive peaks immediately near the plate and as the fluid moves away from the plate, it calms and these peaks slowly decrease.

Velocity distribution for various values of Nr is drawn in Figure 7. It is noticed that for both static plates (λ=0) and moving plate (λ=±1), thermal radiation is a cause of enhancement in fluid velocity as an increase in values of Nr is resulting in elevation of velocity profiles. The physical justification of this increment is the higher rate of energy transport to the fluid. This higher rate results in a reduction of viscous force because the bonds between fluid components get weaker due to the higher energy transport rate. Finally, fluid gets accelerated. Figure 8 covers the effect of solid volume fraction on dimensionless velocity of fluid. It is noticed that fluid flow gets accelerated following an increase in the volume fraction. It is also observed that momentum boundary layer thickness enhances with an increase in ϕ for static (λ=0) and moving (λ=±1) plate. This is due to the factor that increases in ϕ, weakens the viscous forces which leads to a raise in the velocity profile of the fluid. Moreover, Figure 9 demonstrates that for static plate (λ=0) case and moving plate (λ=±1) case, the velocity of the fluid increases with an increase in time *t*. This behavior also explains the transient nature of flow.

In Figure 10, the thermal profile of two types of nanofluids Cu-water and ${\mathrm{TiO}}_{2}$-water are plotted. It is witnessed that the temperature of Cu-water is slightly higher than ${\mathrm{TiO}}_{2}$-water. This difference is supported by the fact that Cu nanoparticles have relatively higher thermal conductivity against ${\mathrm{TiO}}_{2}$ nanoparticles, therefore suspension of Cu in base fluid water enhances the thermal conductivity of Cu-water and eventually temperature of nanofluid rises. It is also observed that the thermal boundary layer thickness is greater in the case of Cu-water. The thermal conductivity of the fluid augmented with Nanoparticle’s addition. These Nanofluids can be considered heat transmission fluids in heat transfer applications. From these temperature profiles, it is anticipated that Cu-water and ${\mathrm{TiO}}_{2}$-water can be used as alternatives in heat exchange processes, under specific conditions. Combination of thermal conductivity and other desirable features such as corrosion resistance and creep rupture strength enable copper to be specified for heat exchangers in industrial field, however, it is expensive and precious when it comes to locating it. Figure 11 demonstrates the behavior of dimensionless temperature for increasing values of Nr. An expected behavior is noticed as the radiation parameter Nr defines the relative contribution of conduction heat transfer to thermal radiation transfer. Hence, it is clear that the temperature will be increased by enhancing the thermal radiation parameter. Physically, $k_{1}$ faces a decay because of elevation in divergence of radiative heat flux $\frac{\partial q_{r}}{\partial y}$. This results in an enhancement in the amount of radiative heat transfer to the fluid, and consequently, the temperature of the fluid rises.

Effect of variation in values of Pr on dimensionless temperature is revealed in Figure 12. It is spotted that nanofluid temperature faces a decay corresponding to increment in Pr. The physical verification of this decay is that fluid with high Pr value has relatively lower thermal conductivity, which, decreases the amount of heat transfer and as a result, temperature reduces. Furthermore, the thickness of the thermal boundary layer decreases. Figure 13 incorporates the influence of heat sink parameter (Q) on the temperature of nanofluid. As expected, an increase in Q results in a decrease of temperature. This is because of the fact that the increase in Q corresponds to more amount of consumed heat which certainly implies that temperature is decreasing function of Q. It can be remarked that heat transfer can be controlled very effectively by including some heat sink in the system.

Figure 14 exhibits the impact of solid volume fraction (ϕ) on thermal profile. Enlargement in ϕ implies enhancement in temperature. It is seen that the temperature of pure water (ϕ=0) is less than the temperature of Cu-water. Moreover, the suspension of nanoparticles in some regular fluid boosts the thermal conductivity of fluid under observation. This physical phenomenon justifies the appreciation in nanofluid’s thermal conductivity corresponding to increasing values of ϕ. Hence, the temperature of fluid increases. It also reveals the meaningful influence of nanofluids in engineering as the processes involving heating and cooling face changes in mass and thermal behaviors due to change in volume fraction of nanoparticles. Figure 15 describes that temperature enhances as the time increases. Physically, plate with relatively higher temperature is exposed to fluid for longer duration, therefore fluid absorbs more amount of heat which leads to enhance the velocity of fluid. Hence, the average kinetic energy of fluid increases and consequently, the temperature of fluid rises. This phenomenon explains the transient effect on heat transfer. Heat transfer augmentation in the attendance of nanoparticles in the base fluid is observed. The convective heat transmission of nanoparticles is dependent on coolant and fluid flow rate. The temperature profile also depicts that near the wall, the temperature is high but it goes to zero gradually as fluid moves far away from the wall. For the validity of the temperature solution of the current problem, a comparative analysis is conducted with [6] in Figure 16. It is clearly observed that both the solutions are in good agreement when the heat sink is removed from the system (Q=0).

**Figure 17:**
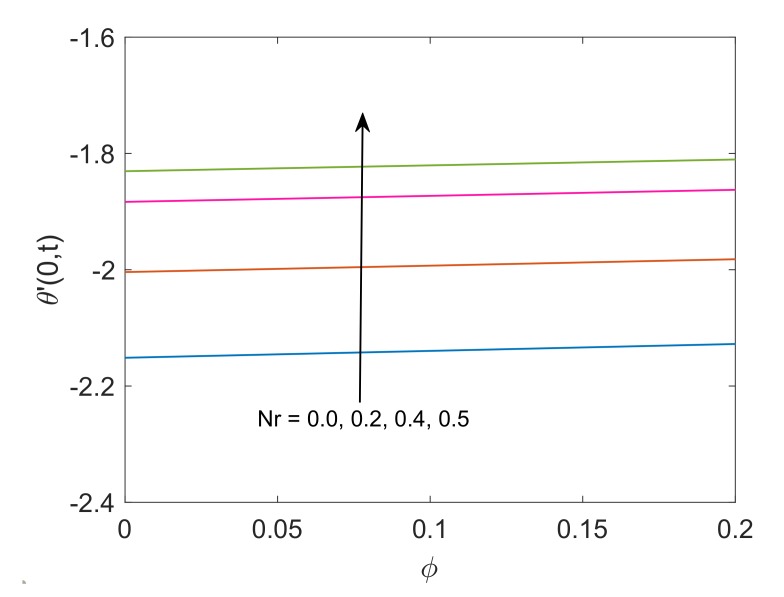
Profile of heat transfer rate for different Nr when Pr=6.2,Q=0.5 and t=0.5.

**Figure 18:**
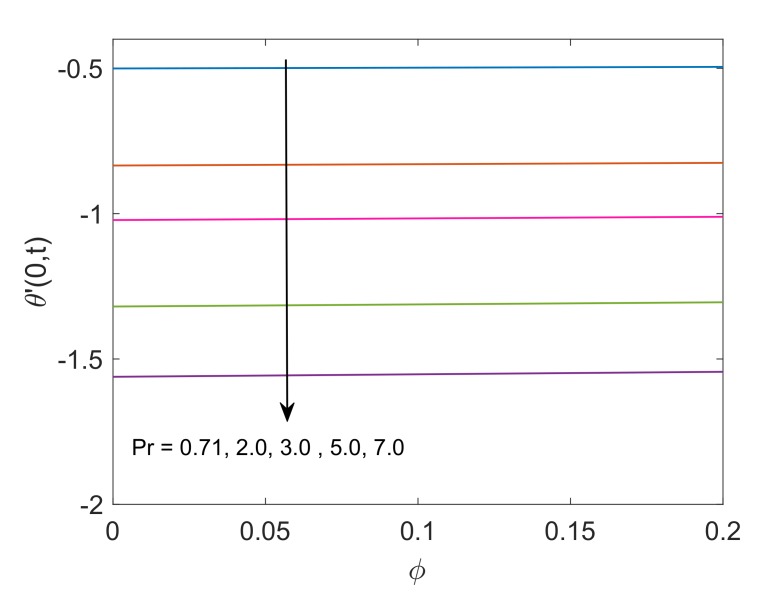
Profile of heat transfer rate for different Pr when Nr=1.5,Q=0.5 and t=0.5.

**Figure 19:**
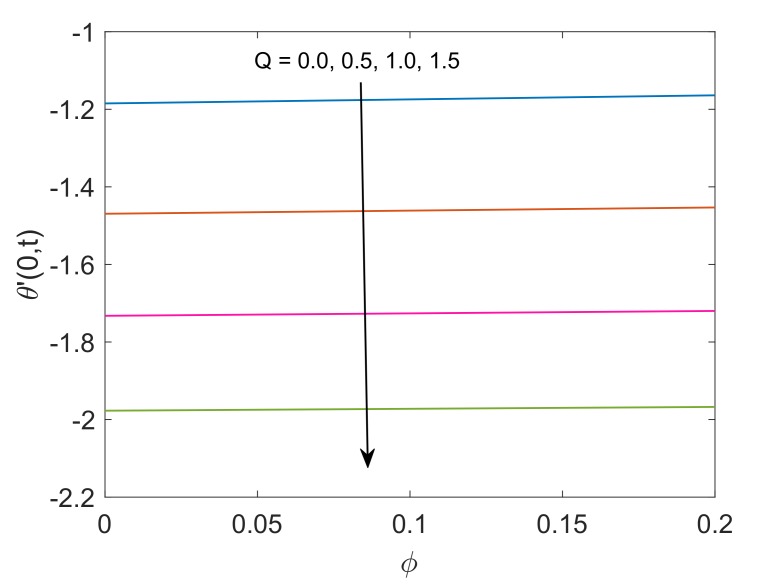
Profile of heat transfer rate for different *Q* when Nr=1.5,Pr=6.2 and t=0.5.

**Figure 20:**
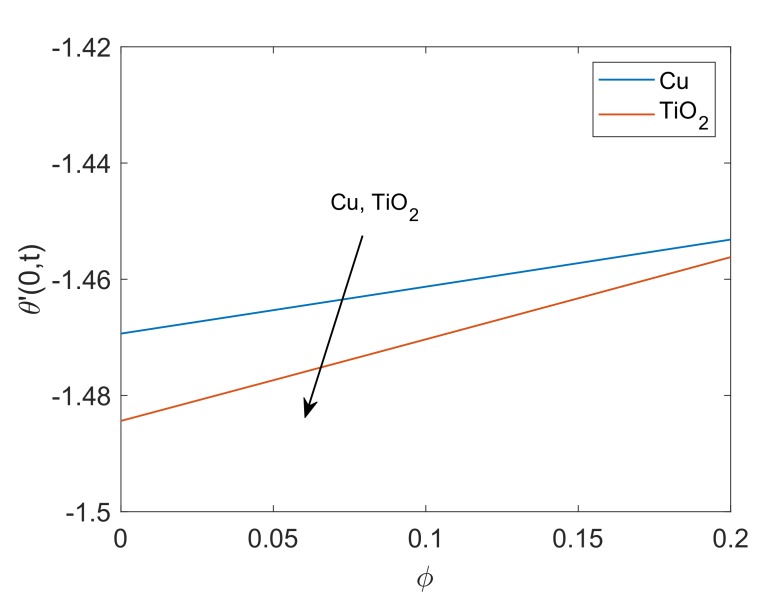
Profile of heat transfer rate for Cu-water and ${\mathrm{TiO}}_{2}$-water when Nr=1.5,Q=0.5 and t=0.5.

Figure 17, Figure 18, Figure 19 and Figure 20 present the variation in heat transfer rate corresponding to several pertinent parameters. Figure 17 reveals that enlargement in Nr values results in the enhancement of the heat transfer rate. This can be justified as the temperature gradient has strong dominance when thermal radiation Nr has higher values. This dominance of temperature gradient increases the rate of heat transfer. Moreover, it is witnessed that with addition in value of ϕ along the x-axis, the heat transfer rate slightly increases. This behavior is supported by the fact that the maximization of solid volume fraction ϕ enhances thermal conductivity and decreases thermal boundary layer thickness. As a result, the greater value of nanoparticle’s volume fraction leads to elevate the rate of heat transfer. The negative sign of the rate of heat transfer shows that the plate is at the receiving end in the process of heat transfer. The reason is that heat is generated near the plate and temperature of fluid may overcome the temperature of the plate. This results in the transfer of heat from fluid to plate.

The control of Pr on the heat transfer rate is graphed in Figure 18. It is noted that heat transfer rate is low for higher Pr values due to the fact that fluid with high Pr values have relatively smaller thermal conductivity, therefore conduction of heat for such fluids is low. This reason concludes that the transfer of heat is low for fluids with greater Pr values. Figure 19 illustrates the heat transfer rate for several values of Q. It is found that the heat transfer rate decreases as the value of Q rises. This behavior is obvious since the heat sink added to the system will absorb the heat, therefore the amount of heat transfer from fluid to plate will be lower. At the end, variation in heat transfer rate regarding different nanofluids are witnessed in Figure 20. It is shown that the rate of heat transfer for Cu-water is higher than that of ${\mathrm{TiO}}_{2}$-water. The fundamental reason behind this comparatively greater rate of heat transfer is the higher thermal conductivity of Cu. The addition of Cu in some base fluid such as water in the current work enhances the thermal conductivity of that fluid, which leads to a greater rate of heat transfer.

Table 1 encloses all the values used in plotting and graphing.

## 5. Conclusions

The aim behind this study is to calculate the exact solutions of time-dependent free convection MHD flow of some nanofluids close to a moving vertical plate, saturated in porous medium incorporating radiative heat flux and heat sink. The non-linear thermal radiation term is linearized by Rosseland approximation. The fundamental partial differential equations along with suitable initial and boundary conditions are made dimensionless first and later Laplace transformation is employed to convert them in ordinary differential equations and solutions are derived in closed form. The meaningful physical contribution of associated parameters in momentum and energy profiles is interpreted with the help of graphs. The expression for Nusselt number is also evaluated to observe the influence of pertinent factors on the process of heat transfer.

The significant results of this study are

- For both nanofluids, the increase in the porosity parameter, magnetic parameter and Grashof number leads to an increase in the velocity of the fluid.
- Temperature of both nanofluids gets elevation with an increase in radiation parameter, while an opposite behavior is noted for increasing values of heat sink parameter.
- Cu-water has greater momentum boundary layer thickness than ${\mathrm{TiO}}_{2}$-water nanofluid.
- Rate of heat transfer increases as the radiation parameter increases, while the increase in values of heat sink parameter reduces the rate of heat transfer.
- ${\mathrm{TiO}}_{2}$-water has a lower rate of heat transfer at the wall in contrast to Cu-water.

## Figures and Tables

**Table 1:** Thermophysical properties of nanoparticles and water [45].

**Fluid/Nanoparticles**	$\rho (\frac{kg}{m^{3}})$	$c_{p}\left(\frac{J}{kgK}\right)$	$k(\frac{W}{mK})$	$\beta \times 10^{5}\left(\frac{1}{K}\right)$	ϕ	σ $\left(\frac{S}{m}\right)$
**Water**	997.1	4179	0.613	21	0.0	$5.5\times 10^{-6}$
**Copper (Cu)**	8933	385	401	1.67	0.05	$59.6\times 10^{6}$
**Titanium Oxide** $\left({\mathrm{TiO}}_{2}\right)$	4250	686.2	8.9538	0.90	0.2	$2.6\times 10^{6}$

## Abbreviations

Gr	Grashof number (-)
Pr	Prandtl number (-)
Q	Parameter of Heat sink (-)
$M^{2}$	Magnetic parameter (-)
Nr	Radiation parameter (-)
Nu	Nusselt number (-)
ρ	Density of fluid (kgm${}^{-3}$)
μ	Dynamic viscosity (Kgm${}^{-1}$s${}^{-1}$)
σ	Electrical conductivity (Sm${}^{-1}$)
ν	Kinematic Viscosity (m${}^{2}$s${}^{-1}$)
*k*	Thermal conductivity (W m${}^{-1}$ K${}^{-1}$)
ϕ	Volume fraction
$c_{p}$	Heat capacitance (J Kg${}^{-1}$K${}^{-1}$)
$B_{0}$	Magnetic field strength (NmA${}^{-1}$)
$k^{*}$	Porosity (-)
g	Standard gravity (ms${}^{-2}$)
$u^{*}$	Velocity (ms${}^{-1}$)
*t*	Time (-)
*y*	Spatial variable (m)
θ	Temperature (-)
L	Laplace transform operator
s	Complex Laplace frequency
K	Parameter of Porosity (-)
$T_{\infty }$	Constant ambient temperature (K)
β	Coefficient of Thermal expansion (K${}^{-1}$)
$k^{*}$	Coefficient of Rosseland adsorption (m${}^{-1}$)
$\sigma^{*}$	Stefan–Boltzmann constant (Wm${}^{-2}$K${}^{-4}$)
